# STRATEGIES AND TOOLS TO MAKE THE INVISIBLE VISIBLE IN HOSPITAL CARE

**DOI:** 10.1093/geroni/igae098.2226

**Published:** 2024-12-31

**Authors:** Blair Golden, Beth Fields, Julia Berian

**Affiliations:** Chair; Co-Chair; Discussant

## Abstract

Family caregivers (relatives, partners, or other support persons) provide critical support during hospitalization events for older adults, ranging from providing companionship and emotional support to coordinating discharge planning. Despite their essential role in hospital care delivery, family caregivers often feel invisible and poorly integrated into hospital care teams. Recognizing this gap, the 2022 National Strategy to Support Family Caregivers was created by federally mandated advisory councils with public input. Goal 2 of this National Strategy consists of advancing partnership and engagement with family caregivers, including recognition of family caregivers as essential care team partners and inclusion of family caregivers within care plans. In response to Goal 2, this symposium will present innovative strategies and tools to advance partnerships and engagement with family caregivers within hospital-based care. Dr. Vick will present findings from a qualitative systematic review of family caregiver involvement during hospitalizations of older adults, leading to development of a conceptual model of family caregiver roles within hospital settings to guide future interventions. Ms. Hoel will discuss findings from a qualitative study identifying organizational barriers to family caregiver inclusion in the hospital care of people living with dementia. Dr. Scheunemann will discuss development of the ICU Family Workbook (a publicly available resource for family caregivers navigating critical illness), including results from content validity and user testing. Dr. Golden will present research findings on current practices and gaps with regards to caregiver and patient discharge education about delirium, a common condition affecting hospitalized adults.

